# Cost-Effectiveness of a Multi-Disciplinary Emergency Consultation System for Suicide Attempts by Drug Overdose in Young People and Adult Populations

**DOI:** 10.3389/fpubh.2021.592770

**Published:** 2021-02-26

**Authors:** Sol I. Kim, Doug Hyun Han, Jin Ho Hwang, Je Hyeok Oh, Myung Hee Shin, Sun Mi Kim

**Affiliations:** ^1^Department of Psychiatry, Chung-Ang University College of Medicine, Seoul, South Korea; ^2^Department of Nephrology, Chung-Ang University College of Medicine, Seoul, South Korea; ^3^Department of Emergency Medicine, Chung-Ang University College of Medicine, Seoul, South Korea; ^4^Department of Safety, Leadership and Coaching, The Graduate School of Psychological Service, Chung-Ang University, Seoul, South Korea

**Keywords:** attempted suicide, drug overdose, emergency psychiatric services, consultation and referral, hospital cost

## Abstract

The purpose of this study was to compare the characteristics of suicide attempts by drug overdose between young people and adults, and evaluate the cost-effectiveness of a multi-disciplinary emergency consultation system (MECS) for suicide attempters with drug overdose. It was verified by comparing and analyzing data from June 1, 2017 to May 31, 2018 (before the MECS was implemented; pre-MECS), and from June 1, 2018 to May 31, 2019 (after the MECS was implemented; post-MECS). The data were retrospectively reviewed for a total of 251 such patients with suicide attempts by drug overdose who visited the emergency room of a university hospital in Seoul during the period. The young people group were shown to be more likely to use painkillers and less likely to use psychoactive drugs for a suicide attempt (*p* < 0.01), had more unplanned attempts than planned ones (*p* < 0.01), and had lower levels of intentionality for suicide (*p* = 0.04) and of suicide lethality (*p* = 0.02), compared to the adult group. We defined suicide attempts as being “serious” when there was both high intentionality and lethality. On this basis, the young people group had less serious suicide attempts, compared to the adult group (*p* = 0.02). Young people in the post-MECS group had lower intensive care unit (ICU) costs (*p* = 0.01) and lower costs in the 6-months after the suicide attempt (*p* = 0.02) compared to those in the pre-MECS group. Young people, both with serious (*p* < 0.01) and non-serious attempts (*p* < 0.01) in the post-MECS group had lower ICU costs compared to those in the pre-MECS group. Adults with non-serious attempts in the post-MECS group had lower ICU costs (*p* < 0.01) compared to those in the pre-MECS group. Therefore, it can be concluded that fast and precise cooperation from the multidisciplinary departments for patients who attempted suicide by drug overdose reduced unnecessary ICU treatment and costs, especially in young attempters and those with lower levels of intentionality and lethality.

## Introduction

Suicide is one of the most common causes of death worldwide and contributes significantly to the global public health cost (1). The World Health Organization (WHO) estimated nearly 800,000 deaths due to suicide in 2016 (2). Intentional self-harm was reported as the most common cause of death under age 70 in South Korea in 2016 (3). South Korea reported the highest suicide rate among the Organization for Economic Cooperation and Development (OECD) countries in 2017, which was 23 per 100,000 South Koreans who died by suicide (2). Medical expenses for individuals who died by suicide in the year before their deaths, and productivity losses from premature deaths were estimated at 3.85 trillion won per year in South Korea (4). Meanwhile, previous suicide attempts are one of the biggest predictors of suicide deaths (5). Around 40% of those who died by suicide have a history of at least one previous attempt (6). Those who had a previous suicide attempt are 12–30% more likely to re-attempt suicide. In addition, there is a higher risk of death by suicide in the first 1–3-years after the initial suicide attempt (7, 8). Therefore, interventions that prevent repeat suicidal behaviors after a suicide attempt are important. There have been studies reporting the benefits of interventions to reduce suicide re-attempts including emergency psychiatric consultation, brief psychotherapeutic interventions, community based case management, and active contact and follow-up interventions using letters/postcards, text messaging, and/or telephone calls (9–11).

For 10–24-years old, defined as “young people” by the WHO (12), suicide is one of the common causes of death (13, 14). According to the WHO definition, young people include both adolescents (10–19-years old) and youth (15–24-years old) (12, 15). Suicide in adolescence consequently reduces the age group of social production, which can adversely affect not only social losses, but also national development. Therefore, early intervention to prevent suicides is necessary (16). A number of results have been published showing differences in the reasons for and methods of suicide attempts between adults and adolescents. Adults often attempt suicide because of psychiatric illnesses, but adolescents are often affected by emotional factors, such as anger, frustration, and rejection in interpersonal relationships (17, 18). Adolescents often seem to choose suicide attempts impulsively as a means of avoiding psychological conflict or expressing extreme pain (19, 20).

Recently, the importance of a crisis intervention system that helps suicide attempters return to everyday life through early management has been emphasized (21). In several countries, various systems are in place, including the operation of special emergency rooms for treating suicide attempters (22, 23). Early detection and management systems for high-risk patients with mental problems in a medical setting, including use of a medical-psychiatry unit or emergency-psychiatry unit, can shorten the duration of hospital stays by reducing delays in psychiatric consultation (21, 22). Therefore, the Ministry of Health and Welfare and the Central Suicide Prevention Center have been operating a project called the “Post-management Service for Suicide Attempters in the Emergency Room” in regional emergency medical centers since 2013.

Taking these into account, a multi-disciplinary emergency consultation system (MECS) for suicide attempters with a drug overdose was established in our hospital. We targeted patients presenting with a drug overdose because it is the most common method of suicide attempt in South Korea (24). The purpose of this study was to compare the characteristics of suicide attempts by drug overdose between young people (10–24-years old) and adults, and evaluate the cost-effectiveness of the MECS for suicide attempters with a drug overdose. We hypothesized that young suicide attempters would tend to have factors associated with more unplanned (impulsive) suicide attempts than planned ones. We also hypothesized that the MECS would reduce unnecessary treatment cost, especially for non-serious unplanned attempts.

## Materials and Methods

### Multi-Disciplinary Emergency Consultation System (MECS)

Initially, before the implementation of the MECS, patients involved in suicide attempts were discharged without psychiatric consultation as it took a long time to respond to consultations, leading to delays or lack of beds in the emergency room. Even when a patient was admitted to another department, a disorganized consultation system resulted in delays in decision-making to transfer or discharge patients and a waste of time. In addition, ambiguous or defensive medical recommendations often made it burdensome for other departments to make decisions. Moreover, integrated care related to the suicidality of patients admitted to various departments and community connections were difficult.

The MECS consisted of internal medicine doctors, emergency medicine doctors, psychiatrists, and social workers. In the MECS, whenever a patient visited the emergency room, the emergency medicine doctor would evaluate physical lethality, ask the psychiatrist to evaluate suicide intention, assign them to the department-in-charge according to their condition, and provide medical care in accordance with prompt consultation from other departments. Also, when a psychiatric consultation was requested after hospitalization in other departments, a time limit of 24 h, excluding holidays, was set to ensure quick responses and treatment decisions. In addition, regular meetings were held every 2 weeks to discuss how suicide attempters could be managed and linked to the community. In the meetings, psychiatrists and social workers were the mainstays for discussing how the community could help the patients, and thereafter, link them to the community mental health and welfare center. The goal of the MECS was to prioritize communication and treatment between departments by evaluating the internal and psychiatric conditions of suicide attempters. It was also devised as a method to shorten the decision time for the transfer or discharge process and to provide clearer and more responsible medical recommendations for each department about the risk of suicide attempt or associated medical conditions.

### Study Participants and Data Acquisition

In this study, the effect of the MECS on suicide attempters with a drug overdose was verified by comparing and analyzing data from the 1-year period between June 1, 2017 and May 31, 2018 (before the MECS was implemented; pre-MECS), and the 1-year period from June 1, 2018 to May 31, 2019 (after the MECS was implemented; post-MECS). The data were retrospectively reviewed for a total of 251 patients who attempted suicide by drug overdose and visited the emergency room of a university hospital in Seoul during the specified period. The age range of our study participants was 13–88-years old; we categorized those aged 13–24 as the “young people” group and those aged 25 or older as the “adult” group. The Institutional Review Board of Chung-Ang University Hospital approved the research protocol. The participants' informed consent was waived because the study was conducted retrospectively and the risk to the participants was considered low.

### Measures

#### Demographic Characteristics

We extracted data on participants' demographic characteristics, including sex, age, years of education, and economic status (monthly household income) from the medical registries for suicidal attempters. Before and after the MECS, we interviewed suicide attempters who visited the emergency room using the initial assessment questionnaire for suicide attempters developed by the Central Suicide Prevention Center. This questionnaire is recommended for use by all hospitals who participated in the project, “Post-management Service for Suicide Attempters in the Emergency Room,” funded by the Ministry of Health and Welfare. The Chung-Ang University Hospital has also participated in this project since 2018 and this questionnaire has been used since then. Data on patients who agreed to participate in this project were maintained in the medical registries for suicidal attempters.

#### Suicide-Related Factors

We extracted data about suicide-related factors from the suicide attempters registration system. These factors include the type of drug used for the suicide attempt (e.g., painkiller/psychoactive medications/herbicides or pesticides), planning for the suicide attempt (planned/unplanned), intentionality of the suicide attempt (intent + behavior/intent – behavior/no intent, no behavior), and lethality of suicide attempts (no physical damage/mild physical damage/moderate physical damage/severe physical damage). The intentionality of suicide attempts was judged by a skilled psychiatrist, and divided into 3 stages: (1) “I really tried to die, and I chose that method” (intent + behavior); (2) “I had a desire to die, but I knew it was not actually a way to die” (intent – behavior); or (3) “I was trying to get help, not really trying to die” (no intent, no behavior). The lethality of suicide attempt was judged by an emergency medicine doctor, and divided into 4 stages: (1) no physical damage, (2) mild physical damage, (3) moderate damage requiring medical attention, and (4) severe damage requiring hospitalization and/or intensive care. In addition, we defined suicide attempts as being serious when both high intentionality corresponding to “intent + behavior” and high lethality corresponding to “moderate to severe physical damage” were present. We defined suicide attempts as being non-serious in all cases that did not correspond to the above criteria.

#### Cost of Hospital Treatment

We extracted data about inpatient expenses for the intensive care unit (ICU costs), inpatient expenses for a general hospital room (general ward costs), and total medical expenses in the 6-months after the suicide attempt (6-month total cost).

### **Statistical** Analyses

The comparison of demographic characteristics, suicide-related factors, and cost of hospital treatment between the pre-MECS and post-MECS groups was conducted using independent *t*-tests and chi-square tests. The comparison of suicide-related factors and hospital costs between the young people and adults groups during the 2-year period, from June 1, 2017 to May 31, 2019, was conducted using independent *t*-tests and chi-square tests. Among the young people and adult groups, the difference in the number of days hospitalized, ICU costs, general ward costs, and 6-month total cost between the pre-MECS and post-MECS groups was analyzed using independent *t*-tests. The difference in the number of days hospitalized, ICU costs, general ward costs, and 6-month total cost between the pre-MECS and post-MECS groups among young people as well as adults with serious and non-serious suicide attempts, were analyzed using independent *t*-tests.

## Results

### Comparison of Demographic Characteristics, Suicide-Related Factors, and Hospital Costs Between Groups

There were no statistically significant differences in sex, age, years of education, and economic status between the pre-MECS and post-MECS groups (Table 1). There were no statistically significant differences in the types of drugs used, planning (planned/unplanned), intentionality, and suicide attempt lethality between the two groups. Further, there were no statistically significant differences in ICU costs, general ward costs, and 6-month total cost between the two groups. However, the number of days hospitalized in the post-MECS group was larger than that observed in the pre-MECS group (*t* = −2.15, *p* = 0.04) (Table 2).

**Table 1:** Demographic data.

	**Pre-MECS** **(*n* = 119)**	**Post-MECS** **(*n* = 132)**	**Statistics**
Sex (male/female)	37 (31.0%)/81 (68.9%)	41(31.1%)/91 (68.9%)	*χ^2^* = 0.02, *p* = 0.89
Age	38.7 ± 19.7	38.2 ± 19.8	*t* = 0.21, *p* = 0.84
**Age grouping**			
Young people (13–24)	39 (32.2%)	50 (37.9%)	*χ^2^* = 0.88, *p* = 0.36
Adults (>25)	80 (62.8%)	82 (62.1%)	
Years of education	10.7 ± 2.8	10.1 ± 2.7	*t* = 1.75, *p* = 0.08
**Economic status ($/year)**			
<20,000	32 (26.9%)	38 (28.8%)	*χ^2^* = 0.95, *p* = 0.62
20,000–40,000	71 (59.7%)	72 (54.5%)	
>40,000	16 (13.4%)	22 (16.7%)	

*MECS, multi-disciplinary emergency consultation system*.

**Table 2:** Comparison of suicidal patterns and hospital costs in all patients.

	**Pre-MECS (*n* = 119)**	**Post-MECS (*n* = 132)**	
**Types of drug**			
Painkiller	12 (10.1%)	21 (15.9%)	*χ^2^* = 2.22, *p* = 0.53
Psychoactive medication	88 (73.9%)	93 (70.5%)	
Herbicide or pesticide	10 (8.4%)	11 (8.3%)	
Other drugs	9 (7.6%)	7 (5.3%)	
Planning for the suicide attempt (planned/unplanned)	45 (37.8%)/74 (62.2%)	43 (32.6%)/89 (67.4%)	*χ^2^* = 0.76, *p* = 0.43
**Intentionality of the suicide attempt**			
Intent + behavior	39 (32.8%)	44 (33.3%)	*χ^2^* = 4.75, *p* = 0.29
Intent – behavior	62 (52.1%)	63 (47.7%)	
No intent, no behavior	18 (15.1%)	25 (19%)	
**Lethality of suicide attempt**			
No physical damage	8 (6.7%)	16 (12.1%)	*χ^2^* = 5.23, *p* = 0.08
Mild physical damage	44 (37.0%)	51 (38.6%)	
Moderate physical damage	35 (29.4%)	39 (29.5)	
Severe physical damage	32 (26.9%)	26 (19.7%)	
Days hospitalized	9.1 ± 8.2	14.7 ± 13.4	*t* = −2.15, *p* = 0.04
ICU costs	2982.9 ± 3092.3	1984.1 ± 2888.7	*t* = 1.33, *p* = 0.19
General ward costs	2484.3 ± 3628.2	3490.1 ± 3880.8	*t* = −1.14, *p* = 0.26
6-month total cost	3069.8 ± 4671.3	2770.4 ± 4779.8	*t* = 0.50, *p* = 0.62

*Psychoactive medications include sleeping pills, antidepressants, anxiolytics, and antipsychotics*.

The intentionality of suicide attempt was divided into 3 stages: (1) “I really tried to die, and I chose that method (intent + behavior)”; (2) “I had a desire to die, but I knew it wasn't actually a way to die (intent – behavior)”; or (3) “I was trying to get help, not really trying to die (no intent, no behavior).”

*MECS, multi-disciplinary emergency consultation system; ICU, Intensive Care Unit*.

### Comparison of Suicide-Related Factors and Hospital Costs Between the Young People and Adult Groups

There was a difference in the sex ratio between the young people and adult groups, such that the proportion of females in the young people group was significantly higher than that of the adult group (Table 3). The young people group used more painkillers to attempt suicide than the adult group (χ^2^ = 16.40, *p* < 0.01), and had more unplanned attempts than planned ones as compared to the adults (χ^2^ = 9.00, *p* < 0.01). The young people group showed lower levels of intentionality for suicide (χ^2^ = 8.36, *p* = 0.04), lower levels of suicide lethality (χ^2^ = 9.41, *p* = 0.02), and less serious suicide attempts (χ^2^ = 5.68, *p* = 0.02), compared to the adult group. In addition, the young people group had lower ICU costs compared to the adult group (*t* = −0.23, *p* = 0.82).

**Table 3:** Comparison of suicidal patterns and hospital costs between the young people and adult groups.

	**Young people (*n* = 91)**	**Adults (*n* = 160)**	
Sex (male/female)	19 (20.9%)/72 (79.1%)	61 (38.1%)/99 (61.8%)	*χ^2^* = 7.95, *p* < 0.01
**Types of drugs**			
Painkillers	24 (24.7%)	11 (6.9%)	*χ^2^* = 16.40, *p* < 0.01
Psychoactive medications	55 (61.8%)	125 (78.1%)	
Herbicides or pesticides	6 (6.7%)	15 (9.4%)	
Other drugs	6 (6.7%)	9 (5.6%)	
Planning for the suicide attempt (planned/unplanned)	21 (23.1%)/707 (76.9%)	67 (41.9%)/93 (58.1%)	*χ^2^* = 9.00, *p* < 0.01
**Intentionality of the suicide attempt**			
Intent + behavior	25 (27.5%)	62 (38.8%)	χ^2^ = 8.36, *p* = 0.04
Intent – behavior	54 (59.3%)	69 (43.1%)	
No intent, no behavior	12 (13.2%)	29 (18.1%)	
**Lethality of suicide attempt**			
No physical damage	7 (7.78%)	16 (10.0%)	*χ^2^* = 9.41, *p* = 0.02
Mild physical damage	37 (40.7%)	43 (26.9%)	
Moderate physical damage	24 (26.4%)	70 (43.8%)	
Severe physical damage	23 (25.3%)	31 (19.4%)	
Serious/non-serious suicide attempt	17 (18.7%)/74 (81.3%)	52 (32.5%)/108 (67.5%)	*χ^2^* = 5.68, *p* = 0.02
Days hospitalized	11.1 ± 10.9	11.7 ± 16.0	*t* = −0.23, *p* = 0.82
ICU costs	1128.9 ± 1527.1	3030.9 ± 3310.8	*t* = −2.56, *p* = 0.01
General ward costs	2131.8 ± 1882.8	3557.7 ± 4655.5	*t* = −1.65, *p* = 0.10
6-month total cost	1794.9 ± 2999.7	1323.9 ± 1913.8	*t* = 0.71, *p* = 0.48

*Psychoactive medications include sleeping pills, antidepressants, anxiolytics, and antipsychotics*.

The intentionality of suicide attempt was divided into 3 stages: (1) “I really tried to die, and I chose that method (intent + behavior)”; (2) “I had a desire to die, but I knew it wasn't actually a way to die (intent – behavior)”; or (3) “I was trying to get help, not really trying to die (no intent, no behavior).”

*MECS, multi-disciplinary emergency consultation system; ICU, Intensive Care Unit*.

Young people in the post-MECS group had lower ICU costs (*t* = 2.67, *p* = 0.01) and lower 6-month total cost (*t* = 2.46, *p* = 0.02) compared to those in the pre-MECS group (Table 4). However, there were no statistically significant differences in the number of days hospitalized and general ward costs between young people in the pre-MECS and post-MECS groups. For adults, there were no statistically significant differences in the number of days hospitalized, ICU costs, general ward costs, and 6-month total cost for those in the pre-MECS and post-MECS groups.

**Table 4:** Changes in the number of days hospitalized and hospital costs between the young people and adult groups.

	**Young people (*****n*** **=** **91)**		**Adults (*****n*** **= 160)**	
	**Pre-MECS (*n* = 41)**	**Post-MECS (*n* = 50)**	**Pre-MECS (*n* = 78)**	**Post-MECS (*n* = 82)**
Days hospitalized	8.24 ± 9.07	13.8 ± 12.1	9.48 ± 7.71	15.62 ± 22.84
ICU costs	2139.4 ± 2191.9^*^	551.6 ± 439.3	3252.8 ± 3322.7	3163.7 ± 3495.1
General ward costs	1519.0 ± 1177.7	2642.6 ± 2218.9	3193.9 ± 4568.2	4337.7 ± 4956.1
6-month total cost	2264.9 ± 3486.3^*^	849.7 ± 116.7	1746.5 ± 5816.7	987.9 ± 1298.4

* *Statistically significant p < 0.05, MECS, multi-disciplinary emergency consultation system; ICU, intensive care unit*.

**Figure 1:**
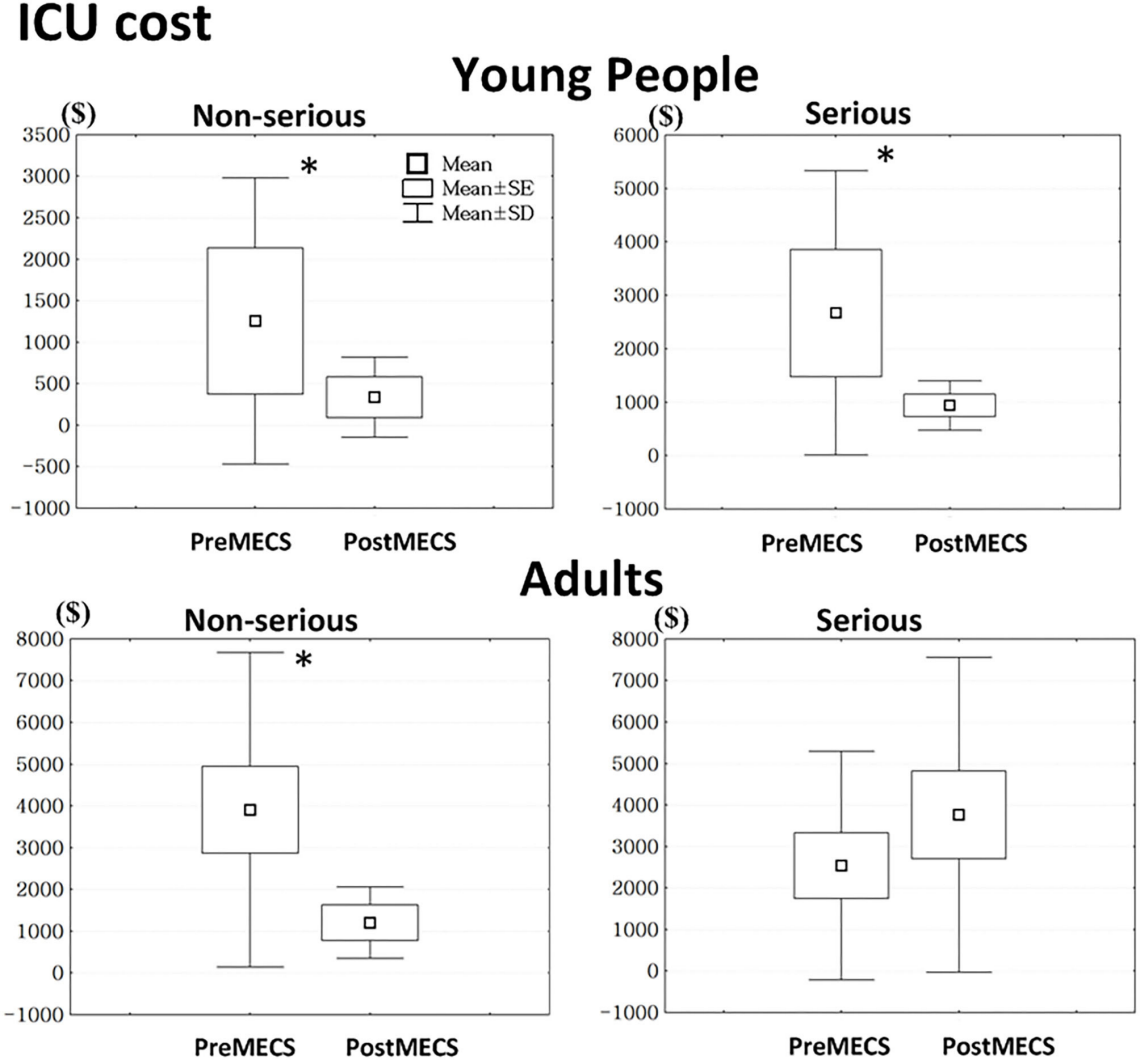
We defined suicide attempts as being serious when showing both high intentionality and high lethality. Otherwise, we defined suicide attempts as non-serious. *Statistically significant, *p* < 0.05. ICU, intensive care unit; MECS, multi-disciplinary emergency consultation system.

Young people with both serious attempts (*t* = −3.17, *p* < 0.01) and non-serious attempts (*t* = −3.23, *p* < 0.01) in the post-MECS group had lower ICU costs compared to those in the pre-MECS group (Figure 1). However, adults with non-serious attempts in the post-MECS group had lower ICU costs (*t* = −4.01, *p* < 0.01) than those in the pre-MECS group.

**Figure 2:**
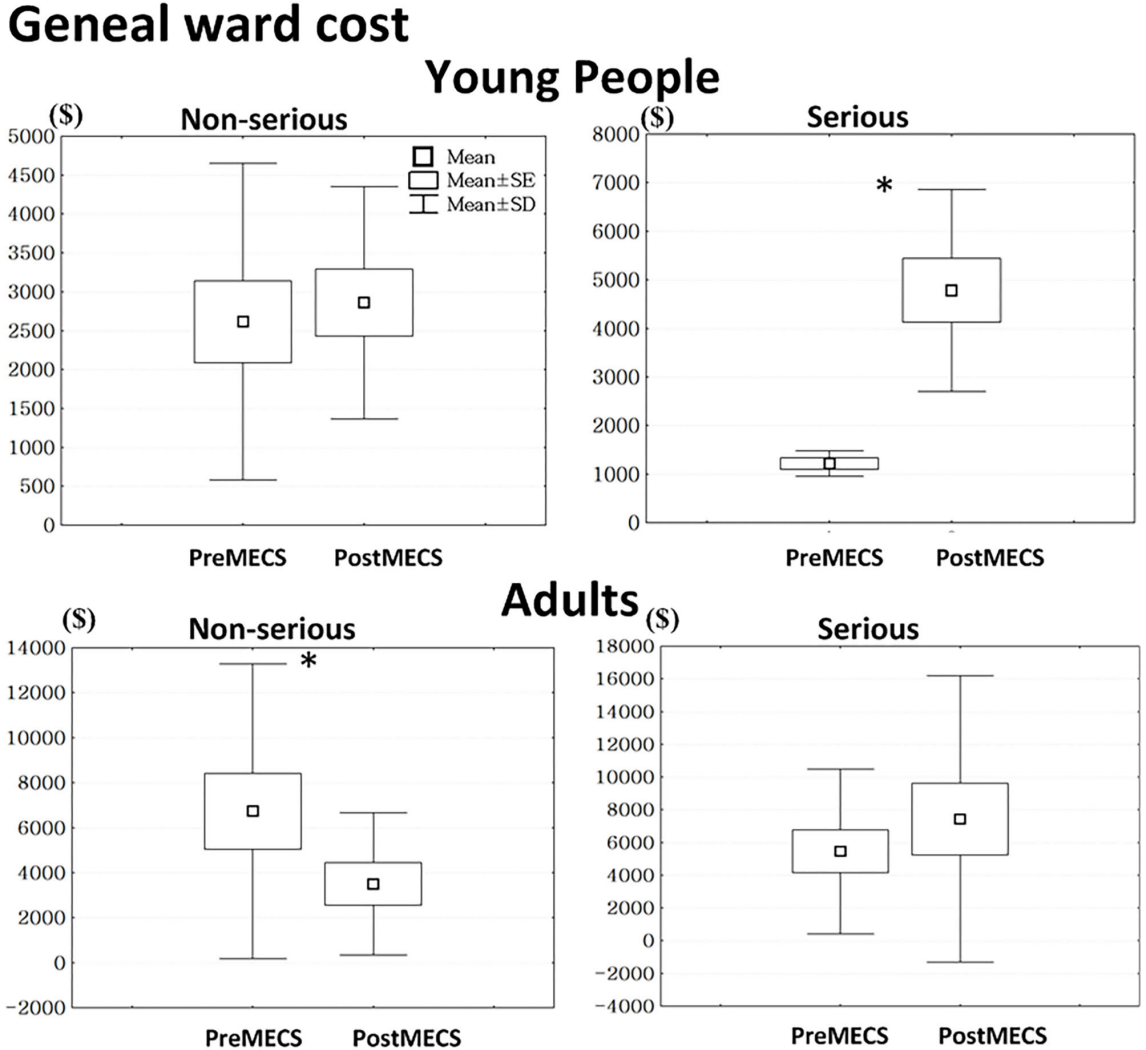
We defined suicide attempts as being serious when showing both high intentionality and high lethality. Otherwise, we defined suicide attempts as non-serious. *Statistically significant, *p* < 0.05, MECS, multi-disciplinary emergency consultation system.

Young people with serious attempts in the post-MECS group had higher general ward costs compared to those in the pre-MECS group (*t* = 4.73, *p* < 0.01) (Figure 2). Adults with non-serious attempts in the post-MECS group had lower general ward costs compared to those in the pre-MECS group (*t* = −2.73, *p* = 0.01).

## Discussion

So far, there have been several cross-sectional studies using the data from the “Post-management Service for Suicide Attempters in the Emergency Room” project (25–29), but few longitudinal studies have been conducted on the effectiveness of post-management services for suicide attempters. Results from the current study show that young people used more painkillers for suicide attempts and had more unplanned attempts than planned ones as compared to the adults. A comparison before and after the MECS implementation revealed that the MECS system was cost-effective, especially for young and non-serious suicide attempters.

In this study, there were differences in the types of drugs used for suicide attempts in young people and adults. Use of painkillers accounted for a higher percentage in young people than in adults, whereas adults tended to use psychoactive medications and lethal drugs, such as herbicides or pesticides. More lethal drug use in adults is in line with results from previous studies, which have reported that patients tend to take deadly drugs for suicide attempts as they get older (30, 31). Another potential reason for the high use of psychoactive drugs in adults is that they may have more access to drugs than teenagers who are under the supervision their parents or caregivers and are limited to psychiatric treatments per their guardian's decisions. There are several possible reasons for the higher use of painkillers among young people compared to adults. Adolescents often find it difficult to get psychiatric treatment, even if they want to, because of stigma about mental health held by their parents (32). Therefore, the availability of psychoactive drugs may be limited for adolescents, which could explain their reasons for choosing more easily accessible drugs like painkillers. Since November 2012, the Ministry of Health and Welfare has implemented a safety-based drug system that allows for the sale of medications, such as painkillers, to increase public access to medication at convenience stores (33). Since then, painkillers have become relatively easy to obtain in South Korea without a prescription (33). Therefore, adolescents may have tended to use over-the-counter drugs instead of psychiatric medications (34).

In this study, young people had a higher rate of unplanned suicide attempts than planned ones, and a lower percentage of intentional suicide attempts than adults, which could be attributed to several factors. According to previous studies, adults often attempt suicide because of psychiatric illnesses, but adolescents are often affected by emotional factors, such as anger, frustration, and rejection in interpersonal relationships (17, 18). Adolescents tend to choose suicide as a means of expressing extreme pain because of emotional factors rather than attempting suicide because they really want to die (19, 20, 35). In fact, adolescents find it difficult to control negative feelings caused by interpersonal problems in family or peer relationships and are likely to engage in non-suicidal self-injury (36). Adolescents are also influenced by the media to a great extent and may imitate celebrity suicides (37, 38). Difficulty with impulse control and immature decision-making processes could also make them vulnerable to internal confusion and external influences, often leading to more unplanned impulsive suicide attempts than planned ones (39). Neurobiologically, this may be related to a lack of sufficient development in the decision-making areas of the brain. The process of controlling impulsiveness and decision-making is deeply related to several parts of the brain, in particular, the pre-frontal cortex (40). The frontal lobe plays an important role in controlling hazardous behavior in relation to various areas of the brain, such as the dorsolateral pre-frontal cortex and the posterior mesofrontal cortex, which control rewarding behavior (41). The gray matter volume of the pre-frontal cortex develops into an inverted U-shape during adolescence (42, 43), and through this frontalization, one can develop the ability to control impulsiveness (44).

In this study, the ICU costs and 6-month total cost for young people were found to decrease, although there was no change in the total treatment costs for adults before and after the MECS implementation. Further, the number of days hospitalized for suicide attempters increased after the MECS implementation. This means that patients received sufficient treatment during the longer period of hospitalization at the same cost for adults and at a lower cost for adolescents after the MECS implementation. In our previous study on the speed and frequency of consultation using the MECS, the time required for a consultation reply after the MECS implementation was significantly reduced (Pre-MECS: 18.39 ± 14.41 h vs. Post-MECS: 11.53 ± 11.00 h), and the proportion of inpatient transfers to the Department of Psychiatry from other departments was significantly increased (Pre-MECS: 12.9% vs. Post-MECS: 36.4%) (45). Similarly, after the MECS implementation, prompt cooperation by the psychiatrist immediately after the physical condition of the patient in the ICU stabilized prevented unnecessary ICU stays, which reduced the ICU cost. Greater reduction in the hospital costs for young people may be because of a higher number of non-serious attempts in young people compared to those in adults. In other words, unnecessary ICU hospitalizations or treatments for suicide attempters reduced, which increased cost-effectiveness. This is consistent with previous studies that showed that psychiatric and general medical management can be provided at the same time for patients in need of psychiatric treatment, through the psychiatry-emergency unit during hospitalization, thereby improving cost efficiency (21, 22). Ensuring proper intervention based on a cooperative system, for patients with both physical and psychiatric problems that are difficult to treat in outpatient clinics, can also have a significant effect on reducing hospital costs (46).

Furthermore, when we compared costs by evaluating non-serious and serious suicide attempters in this study, we found a decrease in ICU costs for both non-serious and serious young people, and non-serious adult suicide attempters. However, in young people, ICU costs reduced whereas general ward costs increased for serious suicide attempters. This may be due to the quick cooperation of the multidisciplinary departments, which reduced unnecessary ICU treatments, especially in the non-serious groups. The internal medicine departments reduced the number of patients who were being hospitalized for unnecessarily long periods in the ICU due to the fear of suicide re-attempts (the ICU is considered safe against suicide re-attempt because of the possibility of quick intervention), or delayed consultation responses. The general ward costs appeared to have increased as patients in the ICU were quickly moved to the general ward for the rest of the necessary treatment. Another explanation for the increase in the general ward costs is that the patients were more likely to be transferred to the psychiatry department and receive long-term care. Meanwhile, evaluate cost-effectiveness, we analyzed inpatient expenses including expense for the ICU, a general hospital room, and total medical expenses over the next 6-months. Additionally, for evaluating hospital management or national policy, resource use after discharge of patients, such as extra-hospital emergency service use, absence from work due to illness, and mortality, can be included for measuring cost-effectiveness (47).

There are a few limitations of this study. First, it was conducted at a single institution. Since it was a tertiary hospital located in a large city, there may be selection errors due to regional restrictions and medical grades. Therefore, further research is needed at hospitals in different regions for longer periods of time to overcome these limitations. Second, this was a retrospective study, and there were limitations in obtaining information about various variables affecting suicide attempts, such as interpersonal relationships, economic status, and professional situations. Additionally, as this study is a retrospective study, for accurately grouping or defining suicide-related factors, such as planning and intentionality of suicide attempts is another limitation. These data were solely based on a previously collected questionnaire intended to be used by all hospitals that participated in the project, “Post-management Service for Suicide Attempters in the Emergency Room.” Further research is needed with a prospective design using a more intensive interview and standard questionnaires to overcome these limitations.

## Conclusion

Fast and precise cooperation from the multidisciplinary departments for patients who attempted suicide by drug overdose reduced unnecessary ICU treatment and costs, especially in young attempters and those with a lower level of intentionality and lethality.

## Data Availability Statement

The data analyzed in this study is subject to the following licenses/restrictions: Due to the retrospective nature of this research, participants of this study did not agree for their data to be shared publicly; therefore, supporting data are not available. Requests to access these datasets should be directed to Sun Mi Kim, sunmikim706@gmail.com.

## Ethics Statement

The studies involving human participants were reviewed and approved by The Institutional Review Board of Chung-Ang University Hospital. Written informed consent from the participants' legal guardian/next of kin was not required to participate in this study in accordance with the national legislation and the institutional requirements.

## Author Contributions

SMK and DHH were responsible for the conceptualization of this study and analyzed and interpreted the data. JHH, SIK, and MHS were responsible for data acquisition. SIK and SMK prepared the initial manuscript. DHH and JHO reviewed and edited the manuscript. All authors have critically reviewed and approved the final manuscript.

## Conflict of Interest

The authors declare that the research was conducted in the absence of any commercial or financial relationships that could be construed as a potential conflict of interest.
